# Histone acetyl transferase GCN5 promotes human hepatocellular carcinoma progression by enhancing AIB1 expression

**DOI:** 10.1186/s13578-016-0114-6

**Published:** 2016-08-02

**Authors:** Sidra Majaz, Zhangwei Tong, Kesong Peng, Wei Wang, Wenjing Ren, Ming Li, Kun Liu, Pingli Mo, Wengang Li, Chundong Yu

**Affiliations:** 1 State Key Laboratory of Cellular Stress Biology, Innovation Center for Cell Signaling Network, School of Life Sciences, Xiamen University, Xiamen, 361102 Fujian China; 2 Xiamen City Key Laboratory of Biliary Tract Diseases, Chenggong Hospital of Xiamen University, Xiamen, China; 3 Department of Pathology, Chenggong Hospital of Xiamen University, Xiamen, China; 4 School of Life Sciences, Engineering Research Center of Molecular Diagnostics, Ministry of Education, Xiamen University, Xiamen, China

**Keywords:** Amplified in breast cancer 1 (AIB1), Cell proliferation, General control non-depressible 5 (GCN5), Hepatocellular carcinoma (HCC), Histone acetyl transferase (HAT)

## Abstract

**Background:**

General control non-depressible 5 (GCN5) is a crucial catalytic component of a transcriptional regulatory complex that plays important roles in cellular functions from cell cycle regulation to DNA damage repair. Although GCN5 has recently been implicated in certain oncogenic roles, its role in liver cancer progression remains vague.

**Results:**

In this study, we report that GCN5 was overexpressed in 17 (54.8 %) of 31 human hepatocellular carcinoma (HCC) specimens. Down-regulation of GCN5 inhibited HCC cell proliferation and xenograft tumor formation. GCN5 knockdown decreased the protein levels of the proliferation marker proliferating cell nuclear antigen (PCNA) and amplified in breast cancer 1 (AIB1), but increased the protein levels of cell cycle inhibitor p21^Cip1/Waf1^ in HepG2 cells. GCN5 regulated AIB1 expression, at least in part, by cooperating with E2F1 to enhance AIB1 transcription. Consistently, GCN5 expression was positively correlated with AIB1 expression in human HCC specimens in two GEO profile datasets.

**Conclusion:**

Since AIB1 plays a promoting role in HCC progression, our results propose that GCN5 promotes HCC progression at least partially by regulating AIB1 expression. This study implicates that GCN5 might be a potential molecular target for HCC diagnosis and treatment.

## Background

Hepatocellular carcinoma (HCC) is the fifth most prevalent cancer and the second most common cause of cancer-related death worldwide [1]. Despite significant progression in diagnosis of HCC, the treatment still lacks clinical efficacy and remains unsatisfactory. Recurrence and metastasis are the main reasons for mortality after the clinical therapy [2], and the 5-year survival rate is limited to mere 30 % [3]. Thus, dissection of the underlying mechanism of HCC progression and identification of potential molecular targets of HCC are crucial.

General control non-depressible 5 (GCN5), is the first identified transcription-related histone acetyl transferase (HAT), and a vital catalytic component of a transcriptional regulatory complex. GCN5 plays an important role in cellular functions, from cell cycle regulation to DNA damage repair [4–6]. Besides normal cellular functions, GCN5 has also been implicated in certain oncogenic roles. c-Myc is often over-expressed in human cancers and is associated with aggressive tumorigenesis and poor prognosis [7]. GCN5 increases the stability of c-Myc protein by acetylating its K323 residue [8, 9]. GCN5 acetylates and stabilizes the translocated E2A-PBX1 oncoprotein in acute lymphoblastic leukemia (ALL) to aberrantly activate HOX, contributing to the failure of cell differentiation [10, 11]. Moreover, GCN5 directly interacts with pygopus homolog 2 (pygo2), a component of Wnt/β-catenin pathway to enhance the growth of breast cancer stem like cells [12]. It has been reported that GCN5 interacts with E2F1 and acetylates H3K9 on its promoter region to facilitate the expression of Cyclin E and Cyclin D1 to promote lung cancer cell proliferation and tumor growth [13]. In breast cancer cells, GCN5 modulates microtubule acetylation mediated by HBXIP (hepatitis B X-interacting protein) oncoprotein and enhances cell migration [14]. Recently, histone acetyltransferase inhibitors are shown to block neuroblastoma cell growth by suppressing GCN5 expression [15], which highlights the impacts of GCN5 inhibitors as potential drugs to treat cancer.

Taken together, these reports indicate a very fundamental role of GCN5 in several types of cancers. Hitherto no conclusive study has been reported that signifies the role of GCN5 in HCC. With such conspicuous role of GCN5 in cellular functions and putative links to cancer, we investigated the role of GCN5 in HCC progression. We found that GCN5 was highly expressed in human HCC tissues and HCC cell lines. Further scrutiny of the role of GCN5 in HCC revealed that GCN5 potentiated HCC progression by enhancing the expression of AIB1, an oncogene that plays a vital promoting role in HCC progression.

## Results

### GCN5 expression is frequently up-regulated in human HCC tissues and cell lines

**Fig. 1:**
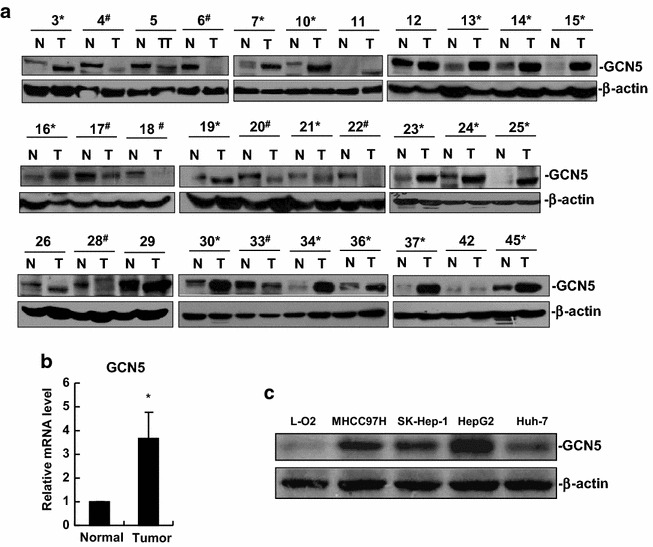
GCN5 expression is frequently up-regulated in human HCC tissues and cell lines. **a** Western blot analysis of GCN5 protein expression in a set of 31 human HCC specimens and surrounding non-tumorous tissues. *N* non-tumorous tissue, *T* tumor tissue. *Asterisk* indicates GCN5 up-regulated HCC specimens. *Hash* indicates GCN5 down-regulated HCC specimens. β-actin was used as a loading control. **b** Relative mRNA levels of GCN5 were up-regulated in HCC specimens. GCN5 mRNA levels in 41 pairs of specimens (tumorous and surrounding non-tumorous liver tissues) were measured by real-time PCR. Relative quantification was achieved by normalization to GAPDH. *p < 0.05. **c** Protein expression of GCN5 was up-regulated in HCC cell lines (MHCC97H, SK-Hep-1, HepG2, and Huh-7) compared with hepatocyte cell line L-O2. All cell lines were cultured in DMEM. β-actin was used as a loading control

To evaluate the involvement of GCN5 in HCC, we examined the expression of GCN5 in a set of 31 human HCC specimens and four different human HCC cell lines by Western blot analysis. Our results showed that GCN5 protein levels were significantly up-regulated in 17 specimens (54.8 %), but down-regulated in 8 specimens (25.8 %), in total of 31 HCC specimens versus the surrounding non-tumorous liver tissues (Fig. 1a). Intriguingly, some GCN5 band shifts were observed (Fig. 1a). In addition, we observed that the mRNA levels of GCN5 were significantly increased in HCC specimens compared with non-tumorous tissues (Fig. 1b).

Furthermore, we analyzed GCN5 expression in human HCC cell lines MHCC97H, Sk-Hep-1, HepG2 and Huh-7 and hepatocyte cell line L-O2. We observed a significant increase in GCN5 expression in HCC cell lines when compared to hepatocyte cell line L-O2 (Fig. 1c). Hence the elevated expression of GCN5 in human HCC specimens and cell lines indicates that GCN5 may be an imperative candidate in HCC progression.

### GCN5 knockdown reduces HCC cell proliferation and colony formation

**Fig. 2:**
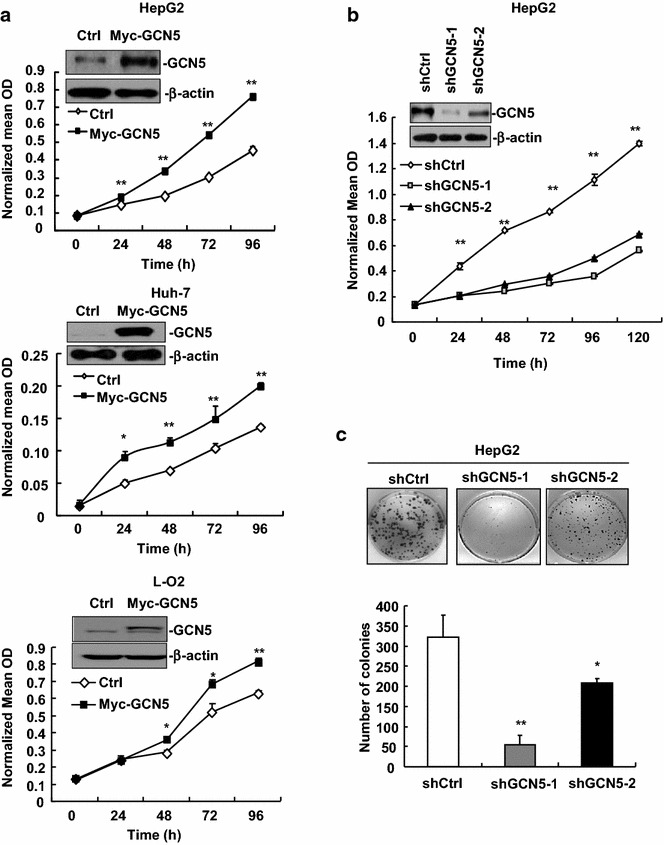
GCN5 knockdown reduces cell proliferation in HCC cell lines. **a** GCN5 overexpression enhanced cell proliferation in HepG2, Huh-7 and L-O2 cells. Overexpression of GCN5 was confirmed by Western blot analysis. MTT assay was performed to determine cell proliferation rate. *p < 0.05, **p < 0.01. **b** Proliferation was reduced in stable GCN5-knockdown HepG2 cells, as measured by MTT assay. Down-regulation of GCN5 was confirmed by Western blot analysis. **c** Knockdown of GCN5 inhibited HCC cell colony formation. *p < 0.05, **p < 0.01

To determine the role of GCN5 in cell proliferation, HCC cell lines HepG2 and Huh-7 cells as well as hepatocyte cell line LO2 were transiently transfected with pCMV-Myc-GCN5 expression plasmids to overexpress GCN5. As shown in Fig. 2a, GCN5 overexpression significantly enhanced the cell proliferation rate of HepG2, Huh-7, and LO2. Furthermore, two stable GCN5-knockdown HepG2 cell lines were established to determine the effects of GCN5 knockdown on cell proliferation. Knockdown of GCN5 significantly decreased the cell proliferation (Fig. 2b), and colony formation (Fig. 2c). These results indicate that GCN5 could potentially promote HCC cell proliferation and colony formation.

### GCN5 knockdown inhibits xenograft tumor formation

**Fig. 3:**
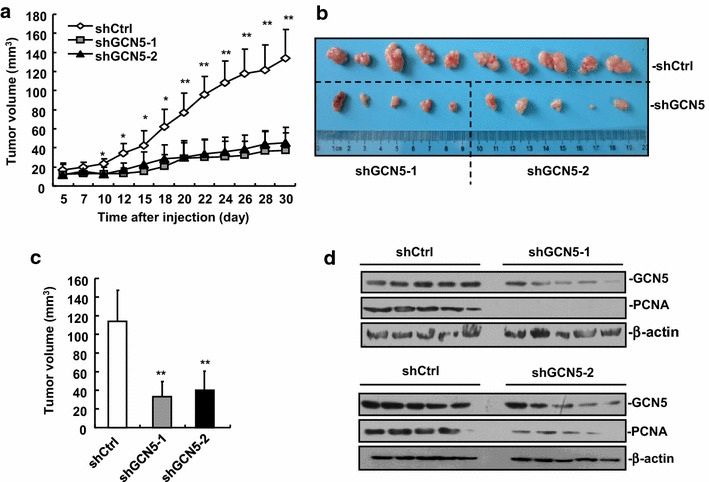
GCN5 knockdown inhibits xenograft tumor formation. **a** Knockdown of GCN5 inhibited HCC cell tumorigenesis in vivo. 1 × 10^6^ HepG2 control cells, shGCN5-1 and shGCN5-2 cells were subcutaneously injected into dorsal flanks of nude mice, respectively. Five days after cell injection, tumor volume was measured every 2 or 3 days. n = 5, *p < 0.05, **p < 0.01. **b** GCN5-knockdown tumors showed a significant decrease in tumor volume compared with control tumors. An image of tumors is shown. **c** Tumor volume was significantly reduced in GCN5-knockdown tumors. **d** The expression of the PCNA was decreased in GCN5-knockdown tumors

To investigate the role of GCN5 in HCC progression in vivo, we assessed the effects of GCN5 knockdown on the growth of HCC xenograft tumors in nude mice. We subcutaneously injected 1 × 10^6^ HepG2 control cells (shCtrl), shGCN5-1 and shGCN5-2 cells in dorsal flanks of five nude mice, respectively. Five days after injection, the tumors were measured every 2 or 3 days for 4 weeks with a Vernier caliper. GCN5-knockdown tumors grew considerably slower as compared to the control tumors (Fig. 3a). At the end of study day (30th) the mice were sacrificed and tumors were excised. The control tumors and shGCN5-knockdown tumors were aligned for comparison. As shown in Fig. 3b, HepG2-shGCN5-1 and shGCN5-2 tumors were much smaller in size as compared to HepG2 control tumors. The tumor volume of GCN5-knockdown group (38 ± 10 mm^3^) was only 34.4 % of the control group (110 ± 30 mm^3^) (Fig. 3c). Consistently, the expression of proliferation marker proliferating cell nuclear antigen (PCNA) was significantly decreased in all representative GCN5-knockdown HepG2 tumors (Fig. 3d). These results suggest that GCN5 plays a key role in HCC tumor growth.

### GCN5 knockdown inhibits cell cycle progression

**Fig. 4:**
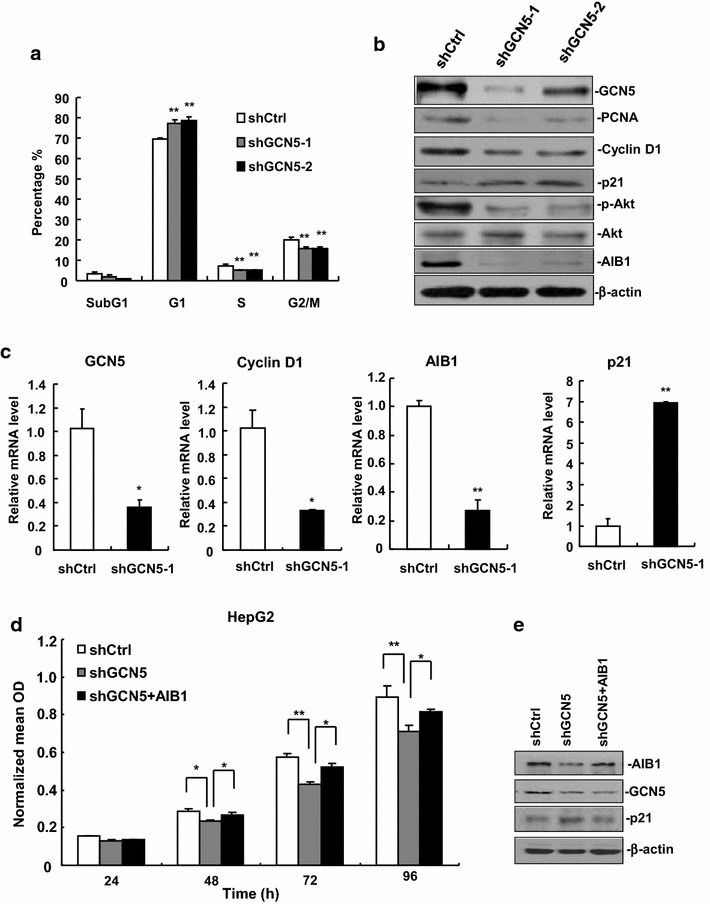
GCN5 knockdown inhibits cell cycle progression. **a** Cell cycle analysis of stable GCN5-knockdown cells and control cells were performed by flow cytometry. A total of 4 × 10^5^ cells were seeded into six-well plates, synchronized by serum starvation for 24 h and re-entered into the cell cycle by an exchange of 10 % FBS DMEM for 24 h. Cell were harvested and cell cycle status was measured by flow cytometry. **p < 0.01. **b** The protein levels of PCNA, p-Akt and AIB1 were decreased, but the protein levels of p21 were increased in GCN5-knockdown cells. **c** The mRNA levels of AIB1 were decreased, but the mRNA levels of p21 were drastically increased in GCN5-knockdown cells. *p < 0.05, **p < 0.01. **d** Forced expression of AIB1 rescued the proliferation of GCN5-knockdown HepG2 cells. Each experiment was performed at least twice with similar results. All data are the means + sd. (n = 3) at each time point. *p < 0.05, **p < 0.01. **e** Forced expression of AIB1 in GCN5 knockdown cells decreased the protein level of p21

Since GCN5 knockdown decreased cell proliferation in HCC cells and attenuated the formation of HCC xenograft tumors in nude mice, we hypothesized that inhibition of HCC proliferation by GCN5 knockdown is due to the cell cycle arrest. To confirm our hypothesis, we analyzed the cell cycle dynamics by flow cytometric analysis. Comparing to the control cells, the cell population in G1 phase was significantly increased in GCN5-knockdown cells, with a corresponding decrease in S and G2/M phases (Fig. 4a). These data suggest that GCN5 is required for the G1-to-S phase transition and its knockdown inhibits the growth of GCN5-knockdown cells by impeding G1/S phase transition of the cell cycle.

To understand the mechanism by which GCN5 knockdown inhibits cell cycle progression, protein levels of several cell cycle-related genes were compared. In two GCN5-knockdown cell lines, while the protein levels of PCNA were significantly decreased as expected, the protein levels of cell cycle inhibitor p21^Cip1/Waf1^ were significantly increased (Fig. 4b). It has been reported that inhibition of Akt signaling can lead to up-regulation of p21^Cip1/Waf1^ [16], and GCN5 can regulate the activation of Akt [17], we therefore detected the protein levels of phosphorylated Akt at Ser473. As shown in Fig. 4b, the expression of phosphorylated Akt at Ser473 was significantly decreased in GCN5-knockdown cells, suggesting that up-regulation of p21^Cip1/Waf1^ is at least in part due to the down-regulation of phosphorylated Akt in GCN5-knockdown cells. AIB1 has been implicated in several cancers [18, 19], and attenuation of AIB1 frequently inhibits the activation of Akt signaling by suppressing the expression of insulin receptor substrate (IRS)-1 and IRS-2 [20–22]. We therefore wondered whether the expression of AIB1 is down-regulated in GCN5-knockdown cells. Our results showed that GCN5 knockdown significantly decreased the protein levels of AIB1 (Fig. 4b). Consistently, GCN5 knockdown significantly decreased the mRNA levels of AIB1, but increased the mRNA levels of p21^Cip1/Waf1^ (Fig. 4c). These results imply that GCN5 promotes cell cycle progression at least in part through up-regulating AIB1 to inhibit p21^Cip1/Waf1^ expression.

To further determine whether GCN5 regulates cell proliferation through AIB1, we performed rescue experiment for proliferation in GCN5-knockdown cells: GCN5-knockdown cells were transfected with AIB1 expression constructs, and then cell proliferation was measured by MTT assay. The results showed that transfection of AIB1 expression constructs could restore cell proliferation potential of GCN5-knockdown cells (Fig. 4d), suggesting that GCN5 promotes HCC cell proliferation at least moderately through regulating AIB1 expression. Western blot analysis of AIB1-restored cells showed a decrease in p21^Cip1/Waf1^ protein expression (Fig. 4e), which further substantiates our results and validates that GCN5 promotes cell cycle progression through up-regulating AIB1 to inhibit p21^Cip1/Waf1^ expression.

### GCN5 regulates AIB1 expression by enhancing de novo transcription of the AIB1 gene

**Fig. 5:**
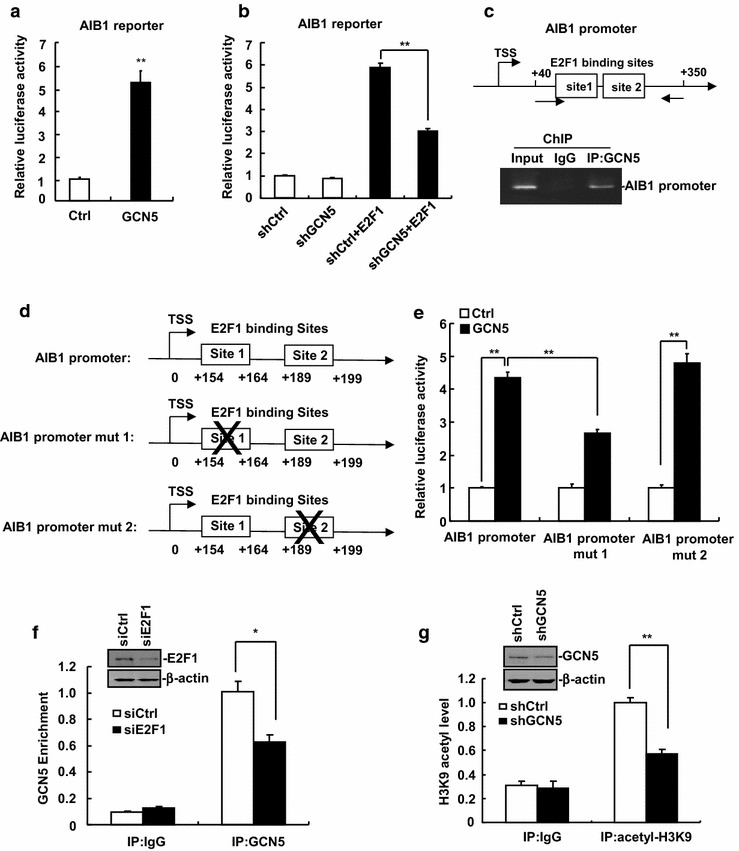
GCN5 regulates AIB1 expression by enhancing de novo transcription of the AIB1 gene. **a** GCN5 enhanced AIB1 promoter activity. 293T cells were transfected with AIB1 promoter reporters with or without PCMV-Myc-GCN5 expression plasmids. Renilla luciferase was transfected as internal control. Luciferase activities were measured and normalized to renilla luciferase value. All data are mean of independent experiments with three replicates per experiments. **p < 0.01. **b** Knockdown of GCN5 significantly decreased AIB1 promoter activity induced by E2F1 transfection. 293T cells were transfected with AIB1 promoter reporters with pGIPZ-shGCN5 plasmids and/or E2F1 expression plasmids. Luciferase activities were measured 48 h after transfection. **p < 0.01. **c** GCN5 was recruited to AIB1 promoter, as measured by ChIP assay. HepG2 cells were lysed for ChIP assays using control IgG and anti-GCN5 antibody for immunoprecipitation. **d** Two E2F1 binding sites were mutated in AIB1 promoter. **e** Mutation of E2F1 binding site 1 (+154 ~ +164 bp) significantly decreased the AIB1 promoter activity induced by GCN5 transfection. Luciferase activities were assayed and normalized to Renilla luciferase activities. **p < 0.01. **f** E2F1 knockdown reduced GCN5 enrichment on AIB1 promoter. siE2F1 was used to knock down E2F1 in HepG2 cells. Cells were lysed for ChIP assays using control IgG and anti-GCN5 antibody for immunoprecipitation. *p < 0.05. **g** GCN5 knockdown reduced H3K9 acetylation of E2F1 binding site on AIB1 promoter as measured by ChIP assay using anti-histone H3 (acetyl K9) antibody for immunoprecipitation. **p < 0.01

Since the mRNA levels of AIB1 were down-regulated in GCN5-knockdown HepG2 cells, we wondered whether GCN5 can regulate de novo transcription of the AIB1 gene. We therefore examined the effect of GCN5 overexpression on the AIB1 promoter activity by using AIB1 promoter reporter assay. Our results showed that GCN5 significantly enhanced the AIB1 promoter activity (Fig. 5a). Unlike a transcription factor, GCN5 protein does not contain a DNA binding domain. Rather than binding to DNA directly, GCN5 is recruited by transcription factors to specific regions of DNA to regulate gene transcription [23]. Because GCN5 has been shown to be a crucial component of E2F-1-transactivating complexes for stimulating E2F-dependent transcription [24], and E2F1 can enhance the AIB1 promoter activity directly [25], we contemplated whether GCN5 is required for E2F1-mediated up-regulation of AIB1 promoter activity. As revealed in Fig. 5b, overexpression of E2F1 significantly enhanced the AIB1 promoter activity as expected, but knockdown of GCN5 significantly decreased the AIB1 promoter activity induced by E2F1 transfection. These results suggest that GCN5 cooperates with E2F1 to enhance de novo transcription of the AIB1 gene.

Consistently, ChIP assay revealed that GCN5 was recruited to AIB1 promoter at E2F1 binding sites (Fig. 5c). Analysis of AIB1 promoter suggested that −250/+350 bp region contains two E2F1 binding sites (Fig. 5d). We performed mutation analysis to determine the role of these E2F1 binding sites in E2F1/GCN5-mediated activation of AIB1 promoter. We mutated E2F1 binding sites-1 and sites-2 on AIB1 promoter and measured AIB1 promoter activity induced by GCN5, respectively (Fig. 5d). Mutation of E2F1 binding site-1 significantly decreased the AIB1 promoter activity induced by GCN5 (Fig. 5e), whereas mutation of E2F1 binding site-2 had no effect on AIB1 promoter activity induced by GCN5 (Fig. 5e). These results suggest that GCN5 is recruited to E2F1 binding site-1 on AIB1 promoter to promote AIB1 expression. To explore whether E2F1 is essential for GCN5 recruitment on AIB1 promoter, we knocked down E2F1 using siRNA and then performed ChIP assay. As shown in Fig. 5f, down-regulation of E2F1 reduced GCN5 enrichment on AIB1 promoter, suggesting that GCN5 is recruited to AIB1 promoter through associating with E2F1.

To determine whether GCN5 is needed to acetylate H3K9 around E2F1 binding site 1 on AIB1 promoter, we knocked down GCN5 and performed ChIP assays using anti-histone H3(acetyl K9) antibody. Our results showed that H3K9 acetylation of E2F1 binding site 1 on AIB1 promoter was significantly reduced in GCN5-knockdown cells as compared to control cells (Fig. 5g), indicating that GCN5 regulates AIB1 expression by acetylating H3K9 around E2F1 binding site 1 on AIB1 promoter.

### The expression of GCN5 positively correlates with AIB1 in human HCC specimens from two GEO profile datasets

**Fig. 6:**
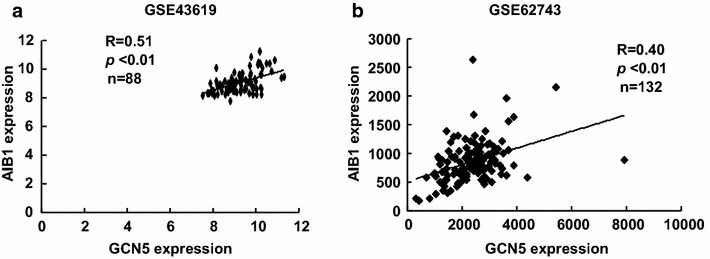
The expression of GCN5 positively correlates with AIB1 in human HCC specimens from two GEO profile datasets. **a** Positive correlation between GCN5 and AIB1 (R = 0.51, *p* < 0.01, n = 88). Expression profiles of 88 human HCC specimens from a GEO dataset (GSE43619). The GCN5 probe ID used was ILMN_1782247, the AIB1 probe ID used was ILMN_1708805. **b** Positive correlation between GCN5 and AIB1 (R = 0.4, *p* < 0.01, n = 132). Expression profiles of 135 human HCC specimens from a GEO dataset (GSE62743). The GCN5 probe ID used was ILMN_1782247, the AIB1 probe ID used was ILMN_1708805

To determine whether the positive correlation between the expression of GCN5 and AIB1 can be verified in a larger cohort of human HCC specimens, we analyzed the expression of GCN5 and AIB1 in human HCC specimens from two GEO profile datasets (GSE41619 and GSE62743). The expression of GCN5 was positively correlated with AIB1 in these two GEO profile datasets (Fig. 6). These results further support our notion that GCN5 regulates AIB1 expression.

**Fig. 7:**
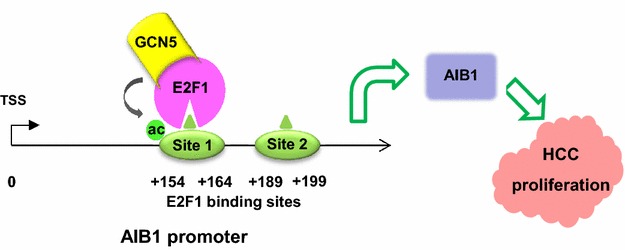
A proposed model illustrating the mechanism by which GCN5 enhances AIB1 expression and promotes proliferation of HCC cells. GCN5 associates with E2F1 and binds to AIB1 promoter to enhance AIB1 transcription by acetylating H3K9 around E2F1 binding site 1 on AIB1 promoter

Collectively, we draft a possible model in which GCN5 enhances HCC proliferation at least partially by enhancing AIB1 expression: GCN5 associates with E2F1 and binds to AIB1 promoter to enhance AIB1 transcription by promoting the H3K9 acetylation on AIB1 promoter, which leads to hyper proliferation of HCC (Fig. 7).

## Discussion

In this study, we demonstrated the key role of GCN5 in HCC progression. GCN5 was up-regulated (54.8 %) in human HCC specimens and in several HCC cell lines, suggesting the involvement of GCN5 in HCC progression. This notion is supported by the fact that GCN5 knockdown hampered the in vitro proliferation of HCC cells and the in vivo growth of HCC tumors in nude mice.

Down-regulation of GCN5 in HepG2 cells resulted in cell cycle arrest. The expression of cell cycle inhibitor p21^Cip1/Waf1^ was significantly increased in GCN5-knockdown HepG2 cells, suggesting that up-regulation of p21^Cip1/Waf1^ at least in part accounts for cell cycle arrest. It has been reported that inhibition of Akt signaling can lead to the up-regulation of p21^Cip1/Waf1^ [16]. In this study, we demonstrated that GCN5 knockdown significantly inhibited Akt signaling by down-regulating p-Akt expression, indicating that up-regulation of p21^Cip1/Waf1^ in GCN5-knockdown HepG2 cells is partly due to the reduced Akt signaling. Our previous study has shown that AIB1 is a bonafide oncogene that is overexpressed in human HCC specimens and promotes HCC progression by enhancing cell proliferation and invasion, and AIB1 knockdown leads to p-Akt down regulation, p21^Cip1/Waf1^ up-regulation and cell cycle arrest in HepG2 cells [26]. Therefore, we speculated that GCN5 knockdown inhibits HCC cell proliferation by down-regulating AIB1 expression. Indeed, knockdown of GCN5 markedly decreased both protein and mRNA levels of AIB1, suggesting that GCN5 promote HCC cell proliferation at least in part by enhancing AIB1 expression.

In this study, we demonstrate that GCN5 can cooperate with E2F1 to enhance AIB1 transcription. Interestingly, it has been shown that AIB1 can also cooperate with E2F1 to enhance gene transcription [25], and E2F1 can stimulate GCN5 transcription [27]. Therefore, it is possible that AIB1 can cooperate with E2F1 to enhance GCN5 transcription. If so, GCN5 and AIB1 may form an amplification circuit to up-regulate each other through cooperating with E2F1, leading to dramatically enhanced cancer cell cycle progression. Further study is needed to validate this possibility.

GCN5 is a histone acetyl transferase (HAT), which acetylates histones to establish an active chromatin environment for transcriptional activation. Inhibition of HAT enzymatic activity by using HAT inhibitors can result in the inhibition of cancer cell proliferation [15]. Several GCN5-specific HAT inhibitors such as CPTH2 have been developed [28, 29]. The effects of GCN5-specific HAT inhibitors on AIB1 expression and HCC cell proliferation are under investigation.

## Conclusions

Since AIB1 plays a promoting role in HCC progression, our results suggest that GCN5 promotes HCC progression at least in part by enhancing AIB1 expression. To our knowledge, this study emphasizes for the first time an important role of GCN5 in HCC progression. Our study leads us toward an additional strategy for targeting AIB1 in several cancers which could provide with new options for combinatorial therapies thus highlighting its significance as a potential therapeutic target for HCC treatment.

## Methods

### Tissue samples and cell lines

Tumorous and adjacent non-tumorous liver tissues were collected from 31 patients who underwent surgery for HCC at the First Affiliated Hospital of Xiamen University. Both tumor and non-tumorous tissues were confirmed histologically. Informed consent was obtained from each patient and the study protocol that conforms to the ethical guidelines of the 1975 Declaration of Helsinki was approved by the Institute Research Ethics Committee, Xiamen University. Human HCC cell lines MHCC97H, SK-Hep-1, HepG2, and Huh-7 and hepatocyte cell line L-O2 were cultured in DMEM supplemented with fetal bovine serum (FBS) and penicillin–streptomycin.

### Cell transfection and luciferase activity assays

The HEK293T cells were transfected with reporter plasmids together with PCR3.1–Rluc as an internal control, in the presence of indicated plasmids by using CaCl_2_ transfection. Cells were harvested 24 h post-transfection and luciferase activity was assayed and normalized to Renilla luciferase activity by using a dual luciferase reporter assay system (Promega, Madison, WI, USA).

### RNA interference and stable cell lines

pGIPZ-shGCN5 plasmids were purchased from Thermo Scientific Company. To generate stably transfected cells, HepG2 cells were transfected with pGIPZ-shGCN5 and control vector, respectively, Approximately 1 week after transfection the cells were treated with 1 μg/ml puromycin for 3 weeks. Five hundred cells were placed in a 100 mm dish and individual drug resistant clones were picked and expanded for further identification. The clones exhibiting significant GCN5 knockdown were used for further experiments. GCN5 specific targeting sequence is TTGAGGGTTGTGTAGAGCT and E2F1 specific targeting sequence is AAGUCACGCUAUGAGACCUCA.

### Cell proliferation

Cell proliferation was analyzed by MTT assay. A total of 3 × 10^3^ cells were seeded into 96-well plates and MTT was added to each well every 24 h. The plates were incubated for 4 h before addition of solubilizing solution (0.01 M HCl in 10 % SDS). The absorbance was measured at 560 nm by using a microplate reader.

### Cell cycle analysis

For cell cycle analysis, 4 × 10^5^ cells were synchronized by serum starvation for 24 h and induced to re-enter the cell cycle by an exchange of 10 % FBS DMEM for 24 h. Both floating and adherent cells were harvested and fixed in 75 % ethanol at 4 °C overnight. Cells were incubated with RNase A at 37  °C for 30 min, and then stained with propidium iodide (PI). Cell cycle was measured by flow cytometry.

### Reverse transcription and real-time PCR

Total RNA was isolated with Trizol reagent (Invitrogen) according to the manufacturer’s instructions. The cDNA was synthesized from 2 μg of total RNA using MMLV transcriptase (Toyobo, Shanghai, China) with random primers. Real-time PCR were performed using SYBR Premix ExTaq (TaKaRa, Dalian, China). Relative quantification was achieved by normalization to the amount of GAPDH. Primers used were: AIB1 forward: GACCGCTTTTACTTCAGGCATT; AIB1 reverse: TGTGTTAACCAGGTCCTCTTGCT; p21 forward: CAGGGGAGCAGGCTGAAG; p21 reverse: GGATTAGGGCTTCCTCTTGG; GAPDH forward: AACTTTGGCATTGTGGAAGG, GAPDH reverse: GGATGCAGGGATGATGATGTTCT; GCN5 forward: GCACAAGACTCTGGCCTTGA; GCN5 reverse: CGGCGTAGGTGAGGAAGTAG; CyclinD1 forward: GTCTGTGCATTTCTGGTTGCA; CyclinD1reverse: GCTGGAAACATGGCCGGTTA.

### Western blot analysis

Equal amounts of protein lysates were separated by SDS-PAGE and transferred onto PVDF membranes. Filters were probed with the following specific primary antibodies: anti-GCN5 (Santa-cruz), anti-AIB1 (BD Bioscience) anti-p21^Cip1/Waf1^ (BD Biosciences), PCNA (Abcam, Cambridge, MA, USA), β-actin (Sigma, St Louis, MO, USA), Akt and phosphorylated-Akt (Cell signaling, Danvers, MA, USA), Anti-histone H3 (acetyl K9) antibody-ChIP Grade (Abcam, Cambridge, MA, USA) anti-CyclinD1 (Abcam, Cambridge, MA, USA). Blots were then incubated with horseradish peroxidase-conjugated secondary antibody (Pierce, Rockford, IL, USA) and visualized by chemiluminescence. The band density was quantified by densitometry using Scion Image software and normalized to β-actin levels.

### Colony formation assays

For focus formation assay, 250 cells were cultured in six-well plates in DMEM with 10 % FBS. After 3 weeks, cells were stained with 0.005 % crystal violet for 1 h to detect foci. Colonies >100 μm in diameter were counted.

### ChIP assay

ChIP assay were processed according to the manufacturer’s instructions. The following primers were used to amplify the DNA fragment corresponding to the sequence from +40 to +350 on AIB1 promoter: forward: 5′-GTCTCAGCCGCTCCACAGCGACGGC-3′ and reverse: 5′-TGAGGGGAAGCGGCGCGGCCCCGAC-3.

### Tumor xenografts

Four to 6-week-old male nude mice were obtained from Laboratory Animal Center of Xiamen University. 1 × 10^6^ HepG2-shGCN5 cells and control cells were subcutaneously injected into the dorsal flanks of mice, respectively. From day 5 after cell injection, the size of the tumor was measured every 2 or 3 days by a vernier caliper along two perpendicular axes. The volume of the tumor was calculated following the formula: Volume = Length × Width^2^ × 0.52. Thirty days after injection, mice were sacrificed and the tumors were photographed and used for western blot analysis. All experimental procedures involving animals were performed in accordance with animal protocols approved by Laboratory Animal Center of Xiamen University.

### Statistical analysis

The data were collected from several independent experiments, with three replicates per experiment. All data were expressed as means + sd. Statistical significant effects (*p* value <0.05) were examined using t test in SPSS 11.0 for Windows (SPSS Inc., Chicago, IL, USA).

## Abbreviations

AIB1: amplified in breast cancer 1
Ch-IP: chromatin immunoprecipitation
FBS: fetal bovine serum
GCN5: general control non-depressible 5
GEO: gene expression omnibus
HAT: histone acetyl transferase
HCC: hepatocellular carcinoma
MTT: 4,5-(dimethyl-2-thiazolyl)-2,5-diphenyl-2-*H*-tetrazolium bromide
PCNA: proliferating cell nuclear antigen
PI: propidium iodide
